# Genome-scale analysis of demographic history and adaptive selection

**DOI:** 10.1007/s13238-013-0004-1

**Published:** 2014-01-30

**Authors:** Qi Wu, Pingping Zheng, Yibu Hu, Fuwen Wei

**Affiliations:** Key Lab of Animal Ecology and Conservation Biology, Institute of Zoology, Chinese Academy of Sciences, Beijing, 100101 China

**Keywords:** genomics, demographic history, local adaptation, natural selection

## Abstract

One of the main topics in population genetics is identification of adaptive selection among populations. For this purpose, population history should be correctly inferred to evaluate the effect of random drift and exclude it in selection identification. With the rapid progress in genomics in the past decade, vast genome-scale variations are available for population genetic analysis, which however requires more sophisticated models to infer species’ demographic history and robust methods to detect local adaptation. Here we aim to review what have been achieved in the fields of demographic modeling and selection detection. We summarize their rationales, implementations, and some classical applications. We also propose that some widely-used methods can be improved in both theoretical and practical aspects in near future.

## Introduction

Identifying adaptive selection has been a central issue in the study of molecular evolution, since Kimura Motoo (1968) argued that it is neutrality instead of selection driving the majority of variations in DNA. There has also been long interest in understanding the nature of selection in study of domestication since Charles Darwin (1859), during which artificial selection leads to phenotypic and genetic variation distinguishing domesticated organisms from their wild ancestors (Mannion, 1999). The inference of demographic history of related population(s) plays a vital role for these aims, for the reason that a proper inferred model could offer a null hypothesis for expectation of neutrality (Nielsen et al., 2007). To distinguish selective traits from those caused by bottleneck effects, understanding of the population history that the first population captured from wild became domestic population has also been a vital task (Axelsson et al., 2013). Besides, demographic models inferred from genetic data complement archeological evidence in understanding pre-historical events, such as number and timing of major continental fluctuations of population size as well as migration. Therefore, the research of demographic history as well as adaptive selection play essential role in evolutionary biology.

In the past decade, there has been an explosive progress in genomics (International Human Genome Sequencing Consortium, 2001; Li et al., 2010; Huo et al., 2012; NCBI Resource Coordinators, 2013). The explosion started from the revolution of sequencing technique and stimulated accumulation of genomic data, which subsequently pushed the improvement of analysis methods. Today, the data accumulation rate is hundreds of times higher than that when the Human Genome Project was first stated (NCBI Resource Coordinators, 2013). The available genomic data have been extended for various species, from the initial goal of human and key lab model species to primates and domesticated animals and plants, and presently to endangered organisms with special scientific or cultural values (NCBI Resource Coordinators, 2013; Grigoriev et al., 2013). Such a data flood made population genetics approaches widely applied in numerous organisms, which in turn stimulated the development of population genetic approaches, for example, to infer more detailed demographic history, to identify adaptive selection more accurately and sensitively, and to perform the computation more rapidly with more data and less constraints (Nielsen et al., 2007; Crisci et al., 2012).

For demographic inference, the most straightforward and simplest approach was based on polymorphic data organized in a Site Frequency Spectrum (SFS). A coalescent process or diffusion process could be applied to trace the history of species. In that way a series of parameters describing the history could be inferred by maximum likelihood, Bayesian approximation or Markov Chain Mento Carlo method (MCMC) (Crisci et al., 2012). As for the identification of local adaptation, population genetic statistic methods were applied to seek the outliers of genetic variation and differentiation across genomes within and between species driven by selection forces (Sabeti et al., 2006). In this review we focused on some recent advances on demographic inference as well as identification of adaptive selection. We also provided some perspectives related to the improvement of those mentioned approaches. We suggested that the genome point of view might contribute to the future progress of population genomics, in both theoretical and applicable aspects.

## Approaches on Demographic History with Genome-Scale Data

To infer demographic history is to estimate population events in the past with population data at present. With the development of the next-generation genome sequencing technique, the present population data could be either mass of genome-scale traditional molecular polymorphism data from multiple individuals or heterozygosity data obtained from one whole genome sequence. We will discuss them respectively.

### Methods with polymorphism dataset

In order to make inference about the population history, two steps are needed. One must firstly formulize the history of the population with certain mathematical model, in which the evolutionary affair during the history was described as a set of parameters. Secondly statistical inference methods could be implied on the parameters. In the past decade, the coalescent process was the most widely used model (Nielsen and Wakeley, 2001; Crisci et al., 2012). Recently, a diffusion approach was adopted as well. These two approaches are discussed in the section of “Coalescent process versus diffusion process”. The statistical inference methods are discussed in the section of “Methods to infer demographic parameters”.

#### Coalescent process versus diffusion process

The isolation-with-migration model (Nielsen and Wakeley, 2001) was one of the most common models to infer demographic scenarios, under which different methods could be used to trace the evolutionary history of the genetic variation. Straightforwardly, the genealogy of alleles could be traced backward in time under the process of coalescence, during which the parameters of demographic model could be derived, including the change of effective population size and the time point on events of bottleneck and exponential growth. When two or more populations were considered, the divergence time could also be derived. If not limited to the consideration of the Wright-Fisher model, coalescent method could consider migration rate and recombination rate in the gene tree as well. Actually, coalescent method is the most widely used method and has been applied in numerous demographic inference programs (Wooding and Rogers, 2002; Adams and Hudson, 2004; Hey and Nielsen, 2004; Thornton and Andolfatto, 2006; Becquet and Przeworski 2007; Lopes et al., 2009; Hey, 2010).

The polymorphism dataset could be organized as the Site Frequency Spectrum (SFS), which is the distribution of allele frequencies in a sampled dataset. In the case of multiple populations, a joint SFS (JSFS) could be used, and the evolution of genetic polymorphism among populations could be described as the change over time of allele distribution in the SFS/JSFS. In the context of neutral theory, the change could be approximated with a diffusion process. The Kolmogorov forward equation for diffusion approximation of neutrality could be introduced to approximate the distribution of allele frequencies at given time (Hartl and Clark, 2007). Different from the methods based on coalescent process, the method based on diffusion process could provide more flexible demographic history model with acceptable computational performance (Gutenkunst et al., 2009), and it has been used to deal with complicated demographic model including three populations with migration and recombination based on genome-scale SNP dataset (Gutenkunst et al., 2009; Zhao et al., 2013).

We would like to review some more about the implementation using the method of diffusion process, not only because the diffusion process has been a classic model in population genetics (Kimura, 1955), but also because it is a distinctive and fairly novel method in demographic inference. A comprehensive implementation named ∂a∂i has been developed until recently (Gutenkunst et al., 2009). Based on the joint distribution of allele frequencies across biallelic variants from multiple populations, the program uses compositional likelihood method to infer expected SFS under a specific demographic model via an evolution process simulated with diffusion. With the assumption of infinite-sites model and Wright-Fisher model, the evolution of density distribution of derived mutations $\Phi \left(x_{1},x_{2},\ldots ,x_{P};t\right)$ in P populations could be formulated as

1 $$\frac{\partial }{\partial \tau }\Phi =\frac{1}{2}\sum_{i=1}^{P}\frac{\partial^{2}}{\partial x^{2}}\frac{x_{i}\left(1-x_{i}\right)}{v_{i}}\Phi -\sum_{i=1}^{P}\frac{\partial }{\partial x_{i}}\left[\gamma_{i}x_{i}\left(1-x_{i}\right)+\sum_{j=1}^{P}M_{i\leftarrow j}\left(x_{j}-x_{i}\right)\right]\Phi$$

Here $\tau =\frac{t}{2N_{ref}}$ is the time unit, where *t* is the time in generations and *N*_*ref*_ is the reference effective population size. *x* means the population frequencies runs from 0 to 1. $v_{i}=\frac{N_{i}}{N_{ref}}$ is the relative effective size of population *i*. *M*_*i* ← *j*_ = 2*N*_*ref*_*m*_*i* ← *j*_ is the scaled migration rate, where *m*_*i* ← *j*_ is the proportion of “chromosomes” per generation in population i that are new migrants from population *j*. And $\gamma_{j}=2N_{ref}s_{i}$ is the scaled selection, in which *s*_*i*_ is the relative selective advantage of variants in population *i*.

The program uses single nucleotide polymorphism (SNP) data in a given genomic region as input dataset, the region of which would be as large as the whole genome obtained by genome resequencing. If outgroup is used, a statistical correction is needed for ancestral state misidentification (Hernandez et al., 2007). Such variations, which is caused by varying mutation rates across sites and over time, violate the parsimony assumption that the ancestral state of each SNP matches the orthologous allele in the outgroup locus (Hwang and Green, 2004). In the original paper of ∂a∂i which inferred a population history of human, a tri-nucleotide transition rate matrix for primate lineage was used for the correction of the misidentification. Since the tri-nucleotide transition rate matrix varies among different mammalian lineages, a customized matrix should be inferred accordingly.

The usage of diffusion approximation offers several advantages of ∂a∂i (Gutenkunst et al., 2009). It considers multiple populations in historical time with population size fluctuations and asymmetric migrations. It goes beyond the assumption of independent non-recombining regions. It uses the full dataset instead of a restricted summary to guarantee the statistical power. Finally and most importantly, ∂a∂i offers a great flexibility to the demographic model design and can be used to model complicated demographic scenarios. With the rapid falling of sequencing cost, ∂a∂i program has been used in several model and non-model species for the whole genome resequencing data. It has been used in soybean to infer domestication history, usually including a bottleneck and a following effective population size fluctuation in the time span of thousand years (Lam et al., 2010). Besides two-population models, ∂a∂i also works well in constructing three-population models as shown in human and giant panda (Gutenkunst et al., 2009; Zhao et al., 2013). In human, a SNP data set of 68 individuals from four populations (YRI, CHB, CEU, and MXL) are used to model human expansion out of Africa and settlement of the New World. To take the out-of-Africa model as an example, three populations, YRI, CHB, CEU, are involved. Population A is the ancestral population. After a population expansion, population B diverges from population A, then split into CEU and CHB with following increases of effective population size respectively (Gutenkunst et al., 2009). In giant panda, a SNP data set of 34 wild individuals from three populations, QIN, MIN, and QXL, are used. The first stage was that ancestral population split into two populations of QIN and non-QIN. Then non-QIN experienced an increase and a following decrease in population size. MIN and QXL split after the decrease of non-QIN, whereas the QIN showed small fluctuations after its split from the ancestor. Besides, a significant asymmetric migration was found from QIN to QXL (Zhao et al., 2013). The brief illustration of the models is shown in Fig. 1.

**Figure 1:**
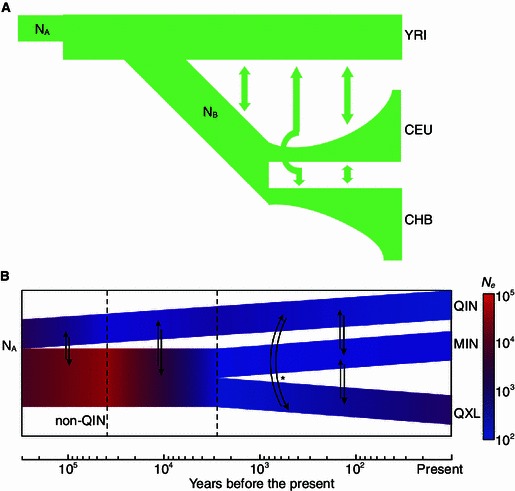
Human and giant panda demographic history inferred by ∂a∂i (See details in Gutenkunst et al. (2009) and Zhao et al. (2013)). Here we focus on the complexity of the two demographic models. Both involve the evolution of three populations (two split from the ancestral population) and changes of the effective population size. (A) The demographic history of three human populations. Population A is the ancestral population whose effective size is shown as N_A_. Population B is the population out of the Africa with effective size N_B_. YRI is the Yoruba individuals from Ibadan, Nigeria. CHB means Han Chinese in Beijing, China. CEU means CEPH Utah residents with European ancestries. (B) The demographic history of three giant panda populations. A is the ancestral population with effective size N_A_. The non-QIN and QIN are divergent populations of population A, and MIN and QXL are divergent populations of non-QIN. QIN and MIN represent the panda population in the Qinling Mountains and Minshan Mountains, respectively. QXL represents a combined population from the Qionglai, Daxiangling, Xiaoxiangling, and Liangshan Mountains. The asterisk in the figure shows the asymmetric migration from QIN to QXL

#### Methods to infer demographic parameters

Demographic history is the population events in the past, while the genetic diversity data available is contemporary. Therefore, one has to find out proper estimation of historical parameters which give the best fit to the present polymorphism dataset. Several alternative statistical inference procedures could be used for these purposes as discussed below. Here we discuss maximum likelihood method and Bayesian approximation method. We also discuss some about Markov Chain Mento Carlo (MCMC) method, which is widely used in Bayesian computation.

In essence, inference of demographic history could be a statistical procedure. It looks for the most possible distribution pattern of SFS under the constraints of given demographic parameter set that fits the real dataset sampled from the population. Therefore, it would be natural to introduce the Maximum Likelihood (ML) method, which estimates the most possible measure of probability in the probability space that fits the known sample with the highest likelihood.

If the sites in the observed dataset are unlike, they could be regarded as statistically independent. Thereby the log-likelihood with the condition of a hypothetical population history model is

2 $$L\left(D|H\right)=\sum_{k=1}^{n-1}S_{k}\ln\sigma_{k}$$

in which $L\left(D|H\right)$ means the log-likelihood of dataset *D* under the condition of population history model, *S*_*k*_ is the number of site occurring *k* times in the sample and *σ*_*k*_ is the probability of a polymorphism sites occurring *k* times in the sample (Wooding and Rogers, 2002). The method has been used to test the fluctuation of effective population size in human population history. In ∂a∂i, the composite likelihood scheme was used (Gutenkunst et al., 2009). With a given distribution of polymorphism of P populations, the expected value of each entry of the SFS was defined as $\text{M}\left[d_{1},d_{2},\ldots ,d_{P}\right]$, where the d is the same as the k in Wooding and Rogers (2002). So the likelihood equation was written as

3 $$L\left(\Theta |S\right)=\prod_{i=1}^{P}\prod_{d_{i}=0}^{n_{i}}\frac{e^{-M\left[d_{1},d_{2},\ldots ,d_{P}\right]}M{\left[d_{1},d_{2},\ldots ,d_{P}\right]}^{S\left[d_{1},d_{2},\ldots ,d_{P}\right]}}{S\left[d_{1},d_{2},\ldots ,d_{P}\right]!}$$

In which $S\left[d_{1},d_{2},\ldots ,d_{P}\right]$ is the joint SFS of the P populations, *L*(*Θ*|*S*) is the likelihood function of the joint SFS under the diffusion model with the parameter set of *Θ*.

The Approximation Bayesian Computation (ABC) is another method to simulate the parameter values from demographic models that could have given rise to the observed dataset. Suppose the observed dataset *x* and the joint density of parameter values *θ* that defined the population history model, the probability of *θ* with given *x* could be considered as a posterior of *p*(*θ*|*x*) according to the Bayesian formula. Thus the essential of the computations become the integral of a certain function of the posterior distribution. The method does work in the case that the posterior distribution was simple or low dimensional, for example, the fluctuation of effective population size on single population (Thornton and Andolfatto, 2006). When the complicated model is considered, the computation becomes a complicated high-dimensional integral which is intractable. Therefore a summary statistics could be used to a restricted set of data to simplify the computation. popABC (Lopes et al., 2009) was following this way, which makes it possible to consider both recombination and migration with genomic data. The program as well as its successive implementations has been used in the detection of rapid radiation in spiny lobsters (Palero et al., 2009), the inference of Africa pygmies demographic history (Batini et al., 2011), as well as the recombination rate variation and the speciation study in rodents (Nachman and Payseur, 2012).

Summary statistics only uses part of information in dataset, which may reduce the statistical power. To use full information of dataset and avoid complicated computation of posterior, the Markov Chain Monte Carlo (MCMC) method was introduced to overcome the difficulty of the complicated high-dimension integral of the posterior in ABC. The method uses a series of sampling based on constructing a Markov chain to get a reliable inference to the probability distribution of the total, instead of computation of high-dimension integral. It starts from settling an initial distribution of the total as prior. Then a series of sampling from the total are performed. For each sampling a distribution could be calculated as the posterior distribution and used to correct the prior one. When sampling times are large enough and the posterior distribution tends to be stable, the stable distribution of samples (exactly the equilibrium distribution of Markov chain) could be considered as the distribution of the total (Beaumont, 2010). The MCMC method was powerful to give a simulation result for the posterior distribution of Bayesian computation particularly for complicated demographic pattern.

The methods for demographic history inference using polymorphism dataset mentioned above were compared in Table 1. From the table it can be seen that majority of the simulation method is coalescent process, that nearly half of the inference procedures is maximum likelihood approach, others is approximation Bayesian computation, and three of the five ABC procedures uses MCMC method. Of the two methods using ML in coalescent process (the first two lines in Table 1), Wooding and Rogers’ method (2002) considered only one population. While Adams and Hudson’s considered two populations. Comparing the two methods using MCMC with full statistics (the line 3 and 4 in Table 1), Hey and Nielsen’s method (2004) considered just two populations while Hey’s extensive method (2010) considered more than two populations.

**Table 1:** Comparison of some recent demographic models

Citations	Simulation method	Inference procedure	Summary statistics	Migration	Recombination
Wooding and Rogers, 2002	Coalescent process	ML	No	No	No
Adams and Hudson, 2004	Coalescent process	ML	No	No	No
Hey and Nielsen, 2004	Coalescent process	MCMC	No	Yes	No
Hey, 2010	Coalescent process	MCMC	No	Yes	No
Thornton and Andolfatto, 2006	Coalescent process	ABC	No	No	Yes
Becquet and Przeworski, 2007	Coalescent process	MCMC	Yes	Yes	Yes
Lopes et al., 2009	Coalescent process	ABC	Yes	Yes	No
Gutenkunst et al., 2009	Diffusion approximation	ML	No	Yes	Yes

#### Model with heterozygosity dataset

Beside above mentioned approaches, another novel approach using heterozygotes of one genome is applied to obtain information of the population parameters (McVean and Cardin, 2005). Such a method, named as the Pairwise Sequentially Markovian Coalescent (PSMC) model, was used to infer human population history (Li and Durbin, 2011). The PSMC model considered the local density of heterozygous sites along chromosomes which reflects how the constant Most Recent Common Ancestors (MRCA, or TMRCA abbreviated by Li and Durbin (2011)) were separated by historical recombination events. Therefore the population parameters, such as the past effective population size and the recombination rate could be inferred.

PSMC deals with heterozygotes in diploid genome. The free parameters of the PSMC model include the scaled mutation rate, the recombination rate, and piecewise constant ancestral population sizes. Indeed, the accuracy and variance of the results depend on the number of recombination events, and very small number of recombination events would increase the variance and reduce the power of the model. Thus too ancient or too recent recombination events would not make a precise inference, because few of these events could be detected in genome. In the original paper of PSMC which inferred a detailed population history from human reference genome, the time span was from 20 Kya to 2 Mya for human demographic history (Li and Durbin, 2011). In the case of bears and giant panda, the time range was 5 Kya to 1 Mya (Miller et al., 2012), and 10 Kya to 9 Mya (Zhao et al., 2013), respectively. For the same species from different populations, the pattern of PSMC fluctuated in a similar way, but skewed a little due to population sub-structure (Li and Durbin, 2011). Fig. 2 showed the results of population history inference in giant panda and human.

**Figure 2:**
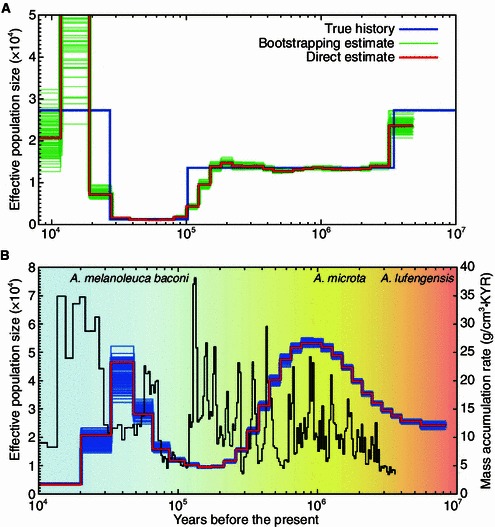
Human and giant panda demographic history inferred by PSMC (See details in Li and Durbin (2011) and Zhao et al. (2013)). (A) The history recovered using human reference genome, which shows a bottle neck during 3–10 Kya following an explosive growth. The red curve is the PSMC estimate on the originally simulated sequence; the 100 thin green curves are the PSMC estimates on 100 sequences randomly resampled from the original sequence. The blue curve is the population-size history of human used in simulation. (B) The history recovered using giant panda genome, in which two peaks of population growth and two bottle necks are detected. The red line represents the estimated effective population size (Ne); the 100 thin blue curves represent the PSMC estimates for 100 sequences randomly resampled from the original sequence; the brown line shows the MAR of Chinese loess which represents the climate changes during the history

## Genome-wide scanning for local adaptation

### From “Survival of the fittest” to “Neutral or near neutral mutations”

The concepts of selection, adaptation, and evolution were first described in the famous Origin of Species by Darwin (1859). Natural selection changes the fitness by accumulating tiny variations from generation to generation to adapt to environments. Phenotypic adaptation is the result of mutation and selection during evolution. A few decades later, Ronald Aylmer Fisher (1930) proposed the idea of “Fisher’s fundamental theorem”, where mathematical approaches were applied to define the fitness rate as a function of its genetic variation. Natural selection could then be measured not only by fitness changes but also by genetic variations.

Later, principles of population genetics were extended to sequence data with the development of molecular biology. The neutral theory considered that genetic variations were accumulation of neutral mutation, and were removed by genetic drift (Kimura, 1968). Successive theories argued that with a broader definition of neutrality, majority of genetic variations could be attributed to random drift during evolution instead of adaptation to local environment (Ohta, 1992; Nei, 2005). Therefore it becomes a challenge to detect the signatures under selection within genome via statistical approaches, which has been one of the central tasks of evolutionary genetics for the recent two decades (Kreitman and Akashi, 1995).

Genome is shaped by two evolutionary forces: neutrality during demographic history and natural selection. Generally, genetic drift, population growth, migration, and other demographic events affect the whole genome; but natural selection by local environment changes make imprint on episodes or a part of structures of the genome, which could have effects on phenotypes and fitness. Natural selection also changes the frequency of mutations across populations. The advantageous mutations approach to genetic fixation under directional selection; the advantageous heterozygotes are maintained by balancing selection; and purifying selection removes the deleterious mutants. The genetic signatures in genome sequence open a door to detect natural selection. Application of mathematical methods and statistical tests throw light on interpreting the imprints of evolution and adaptation.

### Seeking genome-wide signatures of adaptation

Statistical methods are developed to distinguish causal genetic variations subject to selection from neutral genetic variations (Hartl and Clark, 2007). Whole genome sequencing provides large-scale genetic variation data for this purpose. Two strategies are applied. The first one is based on genome-wide selection scans (GWSS) to detect outliers or structure violations as the signatures of selection. The second is based on genome-wide association approaches, in which a prior phenotype or environmental information is required to obtain associated genetic variation loci as the potential candidates for selection. Ultimately, the function of related genes should be investigated to associate the candidate genetic variation with the phenotype variation. For the first strategy, it is a challenge to distinguish selective effect from that caused by neutral events in past. For the second, there are less fine-scaled phenotypic records in non-model species than those in model species or domestic creatures. No matter what strategy is applied, an experimental validation would be highly persuasive for candidate variations, but in most cases it would be very difficult.

#### Comparative genomic data (between/among species)

The simple ratio of non-synonymous (*dN*) to synonymous (*dS*) substitution of coding regions is often used to identify adaptive loci deviation from neutral state between species. Under neutral theory, *ω* = *dN*/*dS* is expected to reflect selection types of species: *ω* = 1, >1, and <1, indicating the sites tested under neutral state, positive selection, and purifying selection, respectively. Comparison of average ω indicator across gene or DNA segments will present the most conservative results because the effects of real sites under selection might be weakened by neutral sites unless the whole region was under selection. Yang (1997, 1998) set varying ω values across lineages or among protein sites to estimate whether a specific lineage was subjected to Darwinian natural selection among protein sites or evolutionary lineages. A likelihood test was applied to determine the null and the alternative hypotheses (Yang, 1997, 1998). This approach was incorporated in PAML software, which included branch model, site model, and branch-site model, allowing ω varying across branches, or sites among proteins, and both across branch and among proteins, respectively (Yang, 1997, 1998; Yang and Nielsen, 2002; Zhang et al., 2005; Yang, 2007; Yang and Nielsen, 2008; Yang and dos Reis, 2011).

These methods have been widely applied in comparative genomic analyses. In mammals, several studies using extensive genomes in mammals have been performed to detect the loci or lineages under selections (Clark et al., 2003; Kosiol et al., 2008; Nielsen et al., 2005; Li et al., 2010). For example, the results of Li et al. (2010) showed that the positively selected genes were significantly enriched in the functional categories of blood circulation and gas exchange activity in mammals. Zhang et al. (2013) compared two related bat genomes and identified genes responsible for DNA damage checkpoint and NF-κB pathways subjected to strong selection, implying possible adaptation to flight. In addition, comparative analysis of two falcons, peregrine and saker falcon, showed an accelerated evolution rate on homeostasis-related genes responsible for circulation (Zhan et al., 2013).

#### Comparative population genomic data (within and between species)

Under the neutral theory, polymorphic and divergence data are expected to be the accumulation of neutral mutations within and between species respectively. Hudson et al. (1987) established a statistical test to examine whether the DNA sequence with higher/lower evolutionary rate between species also presented higher/lower polymorphic rate within species. Although Wright and Charlesworth (2004) extended HKA tests by incorporating maximum likelihood tests, the HKA has been rarely used in genome-wide analysis for its constant effective population size assumption (Nei and Kumar, 2000). McDonald and Kreitman (1991) applied the idea underlying HKA tests and proposed that the ratio of dN:dS between species is equal to the ratio of dN:dS within species when sequences are selective neutrality. They found an excess of the ratio in divergence than that in polymorphism across the ADH sequences among three species of fruit flies, suggesting that beneficial alleles were maintained by positive selection. Bustamante et al. (2005) also applied this approach to compare the divergence and polymorphism data of human and chimpanzee to identify signatures under positive and purifying selection across human genome. The positively selected genes are predicted to be enriched in defense/immunity, transcription, sensory perception and so on, while negative selection are expected to affect processes of cell structure and motility, ectoderm development, general vesicle transport, and intracellular protein traffic.

#### Population genomic data within species

Evolutionary forces will skew genetic diversity and frequency of loci across genomes. Various methods based on estimators of genetic diversity within species have been developed to detect selection signatures. Here we mention tests based on heterozygosity, tests based on *F*_ST_, tests based on Allele frequency, and tests based on haplotype. We also discuss composite approaches combining multiple tests.

Local adaptation signals have two manifestations. Within one population, they are expected to be with low heterozygosity. Between populations they show higher genetic differentiation measured by *F*_ST_. These two indicators, heterozygosity and *F*_ST_, and their derived formations are widely-used in population genomic analysis.

Rubin et al. (2010) applied Z-transformation to pooled heterozygosity $Z\left(H_{\text{p}}\right)$ across sliding windows of the genomes in domesticated chicken, and identified that the loci for thyroid stimulating hormone receptor (*TSHR*) were under selective sweeps. They further tested this gene across 271 birds from 36 geographic populations and proved that the mutant allele is a domestication locus in chicken. The formula they used is as follows.

4 $$H_{p}=\frac{2\times \sum n_{MAX}\times \sum n_{MIN}}{{\left(\sum n_{MAX}+\sum n_{MIN}\right)}^{2}}$$

and

5 $$Z\left(H_{\text{p}}\right)=\frac{H_{\text{P}}-\mu H_{\text{P}}}{\sigma H_{\text{P}}}$$

Genome-wide scanning of adaptation signatures during dog domestication applied the same idea to transform heterozygosity and genetic differentiation index $F_{\text{ST}}$ into $Z\left(H_{\text{p}}\right)$ and $Z\left(F_{\text{ST}}\right)$ between dogs and wolves (Fig. 3), identifying a series of genes in dogs with low $Z\left(H_{\text{p}}\right)$ and high $Z\left(F_{\text{ST}}\right)$, which attributed to adaptation to starch-rich food during domestication (Axelsson et al., 2013).

**Figure 3:**
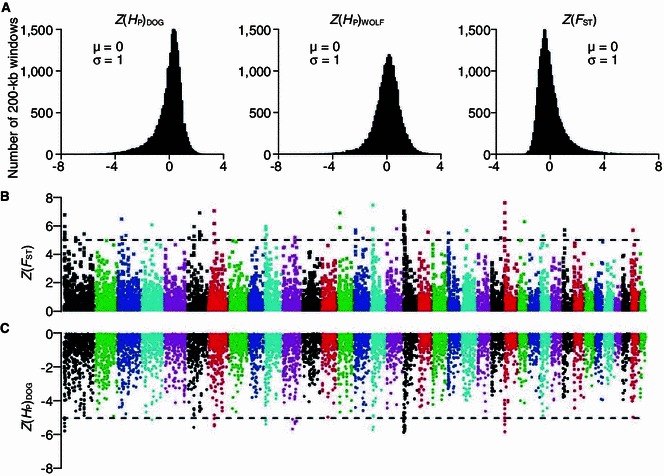
Selection analyses identified 36 candidate domestication regions (cited from Axelsson et al., 2013). Neighbored different colors in (B) and (C) distinguish different chromosomes. (A) Distribution of Z(*H*_p_) of dog and wolf, Z(*F*_ST_) between dog and wolf. (B) Distribution of Z(*F*_ST_) across genomes. The loci with Z(*F*_ST_) over the dashed line cut-off are candidates for outliers. (C) Distribution of Z(*H*_p_) of dogs across genomes. The loci with Z(*H*_p_) value lower than the dashed line are candidate for outliers. The loci with higher Z(*F*_ST_) and lower Z(*H*_p_) values in dogs will be considered as the signatures across genomes under domestication of dogs

Genetic differentiation between populations is usually measured with genetic fixation index $F_{\text{ST}}$. Generally, positive selection gives rise to less heterozygosity within populations and higher genetic differentiation of loci between populations. $F_{\text{ST}}$ statistic has been developed to estimate selection for decades (Wright, 1943; Weir and Cockerham, 1984; Slatkin and Voelm, 1991; Cockerham and Weir, 1993). Pair-wise $F_{\text{ST}}$ between populations is compared to detect the differentiated signals under positive selection directly (Akey et al., 2002; Barreiro et al., 2008; Lam et al., 2010; Zhao et al., 2013). The integrated approach based on different estimation of $F_{\text{ST}}$ has been successfully applied into detection of local adaptation signals in giant pandas population genomics (Zhao et al., 2013).

Yi et al. (2010a) log-transformed $F_{\text{ST}}$ into *T* value:

6 $$\text{T}=-\log\left(1-F_{\text{ST}}\right)$$

They compared the braches of *T* values of Tibetan, Han, and Denes populations to identify signals of Tibetan adapting to high altitudes:

7 $$PBS=\frac{T^{TH}+T^{TD}-T^{HD}}{2}$$

Branch length indicated the genetic differentiation levels. The outliers with larger branches than the average genomic branch in Tibetan were considered to be candidates. To further validate the power of the transformed methods, a larger sample of Tibetan was genotyped, and the frequency of advantageous allele of the outlier loci EPAS1 was examined. The results showed that the SNP in EPAS1 was associated with erythrocyte count and hemoglobin quantity (Yi et al., 2010a).

The proportion of segregating sites in all sites of aligned sequences ${\hat{\theta }}_{S}$ and the average proportion of pairwise mismatches over all compared sequences ${\hat{\theta }}_{\pi }$ could be used to measure nucleotide variation in populations. Tajima (1989) incorporated the two estimators and proposed a statistic test as

8 $$D=\frac{{\hat{\theta }}_{\pi }-{\hat{\theta }}_{S}}{SE\left({\hat{\theta }}_{\pi }-{\hat{\theta }}_{S}\right)}$$

in which *SE* means standard error. Under neutral state, the excess of low-frequency alleles will increase ${\hat{\theta }}_{\pi }$ and that of intermediate-frequency alleles would affect ${\hat{\theta }}_{S}$. A minus *D* value might indicate population under purifying selection or population growth; otherwise, a positive *D* suggests balancing selection, or diversifying selection, and recent admixture of different populations (Hartl and Clark, 2007). Fu and Li (1993) incorporated mutations of external and internal branch to estimate the nucleotide variation, and developed a G statistic test to check the selection signals caused by increased singleton polymorphism. Fay and Wu (2000) proposed an H statistic test to focus on signals from the higher-frequency and intermediate-frequency data. A significant minus H means neutral alleles approached to be fixed for genetic hitchhiking. Among the three statistic methods, Tajima’s D is the most widely used, while the latter two tests incorporated out-group information such as related species to provide ancestral state.

Strong positive selection maintains excess high frequency of beneficial alleles in populations, as shown in Fig. 4. The new advantageous allele relative to its ancestral allele exhibits higher frequency in populations under positive selection. The allele from the related species was taken as the ancestral allele, and the derived alleles were considered the new ones after their divergence. Derived allele of each locus across genomes was compared by simple frequency or relative ratio of frequency among populations to localize the selected new advantageous alleles and its adapted populations, e.g. ΔDAF and DAF tests (Sabeti et al., 2006, 2007; Grossman et al., 2010). The DAF-based tests are expected to identify recent selection events since species divergence. In the example of human and chimpanzee, the derived allele of human SLC24A5 gene shows higher frequency across Europe populations but is absent in most of Asian populations, which is suggested to be responsible to skin pigment difference (Sabeti et al., 2007).

**Figure 4:**
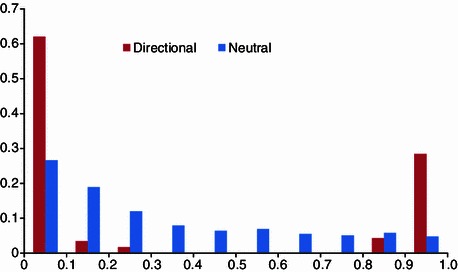
Derived allele frequency spectrum of SNPs under positive selected and neutral selections (modified from Zhao et al., 2013). Positive selection maintained an excess of lower and higher frequency of alleles within populations

Haplotype provides integrative information of a set of neighboring SNPs rather than a sum of individual SNPs. Diversity and frequency of haplotype in populations will be distorted under very recent positive selection. Since the phased high-resolution human HapMap was available, statistic methods based on variance in heterozygosity, length and frequency of haplotype were developed to detect very recent adaptation within and between populations (Sabeti et al., 2002, 2006; Voight et al., 2006). Extended haplotype homozygosity (EHH test) was applied to identify long-range haplotype with reduced heterozygosity as signatures under recent selective sweeps (Sabeti et al., 2002). The ancestral and derived haplotypes were compared using integrated Haplotype Score (iHS test) to measure the selection strength of each locus (Sabeti et al., 2006; Voight et al., 2006). Differential frequency of haplotype between populations was measured and compared in Cross Population EHH (XP-EHH test) to localize population adaptive to local environment (Sabeti et al., 2007; Grossman et al., 2010). The haplotype-based methods were also widely used in domesticated species to detect signals of local adaptation to new environments under artificial selection. Vonholdt et al. (2010) used *F*_ST_ and XP-EHH methods to identify several SNPs involving memory formation and behavioral sensitization during dog’s domestication. Toomajian et al. (2006) proposed a haplotype-sharing statistical analysis and used another haplotype-based method EHH as well to identify the early-flowering alleles in *Arabidopsis thaliana.*

To get a reliable result, it is persuasive to combine multiple tests based on different assumptions for the dataset, and only the outliers supported by multiple tests are considered as reliable candidates (Simonson et al., 2010; Zhao et al., 2013). An alternative approach is to seek the genomic regions with multiple contiguous outlier loci as the candidates under selective sweep (Li et al., 2010). However, Grossman et al. (2010) proposed a composite likelihood test (CMS) incorporating as many as six independent statistic methods (*F*_ST_, ΔDAF, DAF, XP-EHH, iHS, IHH) to identify truly selected variants. A SNP locus was estimated to be with a probability of selected / unselected of each independent test (*Si*). Bayesian factor and CMS were calculated as follows:

9 $$BF=\prod_{i=1}^{n}\frac{P\left(S_{i}|selected\right)}{P\left(S_{i}|neutral\right)}$$

10 $$CMS=\prod_{i=1}^{n}\frac{P\left(S_{i}|selected\right)}{P\left(S_{i}|selected\right)\times \pi +P\left(S_{i}|neutral\right)\times \left(1-\pi \right)}$$

in which the Greek π was the prior probability of selection, being estimated from prior information of the data. The distribution of posterior probability of CMS scores was used to estimate the confidence intervals. A modified CMS test was applied to detect region within genomes for recent adaptation in human populations (Grossman et al., 2013). The authors applied statistical methods to prove that the CMS tests have more power in detection of adaptive signals than independent test (Grossman et al., 2010, 2013).

### Functional analysis of candidates

Generally, three approaches are used to investigate biological function of outlier candidates. One is the prediction of potential gene function. For protein within the candidate selective locus, the spatial structure of the protein will be simulated to infer its potential conformation change influenced by the variant locus (Sabeti et al., 2007; Grossman et al., 2013). For all the outlier data, candidate loci will be annotated by gene ontology database and the functional categories enriched with candidate loci would be considered as potentially selected. An alternative precise functional analysis associates studies of outlier variations with phenotype variation or environmental change. Some researchers have made good examples on genome-wide selection scanning as well as genome-wide association analysis (Yi et al., 2010; Grossman et al., 2013).

However, the prediction analysis and statistical association analysis could only tell us about the general information about potential biological function, it’s important to examine whether the genetic variations with adaptive selection signals successfully express the phenotype with high fitness into new environment or not. In dog, expression and functional experiments were performed after the GWWS analysis to test adaptive signatures under domestication, which proved that domesticated dogs have more powers in enzymatic activity for digestion of starch-rich diets than their ancestor wolves (Axelsson et al., 2013).However, few cases could examine the functions of polymorphic loci in inter-gene under selections because of no protein expression.

## Perspectives

### A diffusion approximation model with recombination

The model used in ∂a∂i could give very complicated demographic model with the least constraints on dataset. The Kolmogorov forward equation, based on Fokker-Planck equation, was very solid in mathematics as well as in population genetics (Kimura, 1955; Haken, 1983). One possible improvement of the model might include the consideration of recombination. Here we suggest a concept of “loci group” to discuss a possible method considering recombination in the diffusion process of polymorphism distribution among populations. In ∂a∂i, all derived mutations from one genome are put into one pool or group to get a density distribution, which could be used to infer demographic history. Thus a prerequisite of unlinked loci within the whole group is needed and the recombination events between any two mutations could not be considered. Here we suggest an improvement that considers two such groups, group A and group B. Both of them satisfy the condition of performing demographic inference following the approach of ∂a∂i. Besides, each SNP in one group has one and only one close-linked locus in the other group. We could say the two groups are to some extent “linked”. For group A, the evolution of the density could be modeled following ∂a∂i’s approach as

11 $$\frac{\partial }{\partial \tau }\Phi_{\text{A}}=\frac{1}{2}\sum_{i=1}^{P}\frac{\partial^{2}}{\partial x_{A}^{2}}\frac{x_{Ai}\left(1-x_{Ai}\right)}{v_{Ai}}\Phi_{\text{A}}-\sum_{i=1}^{P}\frac{\partial }{\partial x_{Ai}}\left[\gamma_{Ai}x_{Ai}\left(1-x_{Ai}\right)+\sum_{j=1}^{P}M_{A:i\leftarrow j}\left(x_{Aj}-x_{Ai}\right)\right]\Phi_{\text{A}}$$

Similarly for group B, one has

12 $$\frac{\partial }{\partial \tau }\Phi_{\text{B}}=\frac{1}{2}\sum_{i=1}^{P}\frac{\partial^{2}}{\partial x_{B}^{2}}\frac{x_{Bi}\left(1-x_{Bi}\right)}{v_{Bi}}\Phi_{\text{B}}-\sum_{i=1}^{P}\frac{\partial }{\partial x_{Bi}}\left[\gamma_{Bi}x_{Bi}\left(1-x_{Bi}\right)+\sum_{j=1}^{P}M_{B:i\leftarrow j}\left(x_{Bj}-x_{Bi}\right)\right]\Phi_{\text{B}}$$

Since the group A and B are from the same genomes and experienced the same history for the same populations. Then we have

13 $$\frac{\partial }{\partial \tau }\Phi_{\text{A}}=\frac{\partial }{\partial \tau }\Phi_{\text{B}}$$

The $\Phi_{\text{A}}$ and $\Phi_{\text{B}}$ should be influenced by the same migration affairs in history. Suppose there is no selection (*γ*_*i*_ = 0), we should have

14 $$\sum_{j=1}^{P}M_{B:i\leftarrow j}\left(x_{Bj}-x_{Bi}\right)=\sum_{j=1}^{P}M_{A:i\leftarrow j}\left(x_{Aj}-x_{Ai}\right)$$

Equations (13) and (14) determine that the drift terms in (11) and (12) should also be equal

15 $$\frac{x_{Ai}\left(1-x_{Ai}\right)}{v_{Ai}}=\frac{x_{Bi}\left(1-x_{Bi}\right)}{v_{Bi}}$$

That means, if there is a certain proportion of chromosomes transformed from population *i* to *j* per generation detected in group A, there could be expected the same proportion in group B. However, if recombination exists, the linkage between A and B might be broken in a probability $r_{i\leftarrow j}$, the recombination rate for one migration event from *j* population to *i* population. In this case, although one allele from group A was migrated, its linked allele in group B was left in the original population in the probability of $r_{i\leftarrow j}$. Therefore one has

16 $$\sum_{j=1}^{P}M_{B:i\leftarrow j}\left(x_{Bj}-x_{Bi}\right)=\sum_{j=1}^{P}M_{A:i\leftarrow j}\left(1-r_{i\leftarrow j}\right)\left(x_{Bj}-x_{Bi}\right)$$

Then the recombination rate could be included in the extensive diffusion process of the density distribution *Φ* as

17 $$\begin{array}{cc} \frac{\partial }{\partial \tau }\Phi & =\frac{1}{2}\sum_{i=1}^{P}\frac{\partial^{2}}{\partial x_{A}^{2}}\frac{x_{Ai}\left(1-x_{Ai}\right)}{v_{Ai}}\Phi +\frac{1}{2}\sum_{i=1}^{P}\frac{\partial^{2}}{\partial x_{B}^{2}}\frac{x_{Ai}\left(1-x_{Ai}\right)}{v_{Ai}}\Phi \\ & \;+\sum_{\text{i}=1}^{P}\frac{\partial^{2}}{\partial x_{A}\partial x_{B}}\left[\sum_{j=1}^{P}M_{A:i\leftarrow j}\left(x_{Aj}-x_{Ai}\right)\right]\left[\sum_{j=1}^{P}M_{A:i\leftarrow j}\left(1-r_{i\leftarrow j}\right)\left(x_{Bj}-x_{Bi}\right)\right]\Phi \\ & \;-\sum_{i=1}^{P}\frac{\partial }{\partial x_{Ai}}\sum_{j=1}^{P}M_{A:i\leftarrow j}\left(x_{Aj}-x_{Ai}\right)\Phi -\sum_{i=1}^{P}\frac{\partial }{\partial x_{Bi}}\sum_{j=1}^{P}M_{A:i\leftarrow j}\left(1-r_{i\leftarrow j}\right)\left(x_{Bj}-x_{Bi}\right)\Phi \end{array}$$

For total *P* populations there will be $\text{P}\times \left(1-\text{P}\right)$ number of *r* values, their mean value could represent the recombination rate of the P populations within one generation. In the whole process, the most urgent task is to determine the SNP pairs. One pair of SNPs should be tightly linked so that there is only one recombination event could happen between the SNPs, as the same time every two pairs of SNPs should be distant enough so that the linkage could be ignored within groups. However, there is an arbitrary assumption which may be violated in real data set in the model. We suppose that to make sure equation (13) is equal, every term in the diffusion function (11) and (12) are equal. Therefore we obtain equation (14) and (15), which mean migration and drift are equal between groups. However, there does be possibility that drift and migration have similar effects, therefore equation (14) and (15) are not equal but equation (13) is still satisfied. If so, a deviation may appear in the estimation of recombination rate. Future studies are needed to access the deviation by either simulation or real dataset.

### Challenges for identifying genomic signature of selection

In genome era, selection tests for recent positive selection were well developed as shown above. However, the test for the balancing and negative selection within species is rare. Although some approaches and softwares made efforts to test balancing selection (Excoffier et al., 2009; Excoffier and Lischer, 2010) and negative selection (Tajima, 1989; Yang, 1998), it could not meet the needs for GWWS, e.g. few balancing and purifying selection tests based on haplotypes. Signatures for balancing and negative selection should be interpreted and mined from large-scale genome data, which calls for diversifying methods in the future.

Genomic signatures for selection detected by GWWS could be distorted by demographic history and population structure. Tajima’s D could not distinguish the signatures caused by selection force from those caused by demographic fluctuation (Yang, 2006, Hartl and Clark, 2007). Other estimators are also sensitive to population growth and contraction (Hartl and Clark, 2007), and invisible population structure and admixture will influence the heterozygosity and haplotype diversity (Hartl and Clark, 2007). Hence it is important to infer population genetic structure and demographic background to distinguish the effect of selection from neutrality. Williamson et al. (2005) set a good example on this idea. He used presumed neutral loci to construct a population history. And then he used the simulation results as the null hypothesis to infer selection signals. The more genomic data available, the more intensive and integrative methods are needed in the future to incorporate both selection forces and demographic history influences.

Last but not least, it is still a barricade to test the selective effect on certain loci using experimental approaches, either for cellular and molecular approach or high-throughput technique. The difficulties come from two aspects. Firstly, it is still a hard task to avoid false negative results. The theoretical assumption may be violated in the populations of certain species, genetic structure or demographic history may not be totally excluded. Selection signals may be flooded by noise. All these may introduce false negative results. Secondly, present methods could give evidence of whether one locus was under selection, but could not give clues that in which levels the locus shows the selection: at molecular level, or cellular level, or tissue/organ level, or individual level. It is impossible to test every possible level for each loci, let alone the permutation and combination of all loci. In general, although it is easy to consider some possibilities that the loci in non-synonymous sites would cause variations in protein sequence and possibly cause the change of protein structure, to verify the biological effects of the identified selective loci will lag behind the progress of identifying selective loci in the next several years.

## Abbreviations

CMS: composite likelihood test
EHH: Extended haplotype homozygosity
GWSS: genome-wide selection scans
iHS: integrated Haplotype Score
MCMC: Markov Chain Mento Carlo method
ML: maximum likelihood
MRCA: Most Recent Common Ancestors
PSMC: Pairwise Sequentially Markovian Coalescent
SFS: Site Frequency Spectrum
SNP: single nucleotide polymorphism
TSHR: thyroid stimulating hormone receptor
